# Health Economic Assessment of PM_2.5_ Originating from Residential Wood Combustion—A Case Study in Northern Sweden

**DOI:** 10.3390/ijerph23081053

**Published:** 2026-08-13

**Authors:** Johan Sommar, Joseph Spadaro, Christian Asker, Hans Orru

**Affiliations:** 1 Department of Epidemiology and Global Health, Umeå University, SE 901 87 Umea, Sweden; hans.orru@ut.ee; 2 Spadaro Environmental Research Consultants (SERC), Philadelphia, PA 19142, USA; spadarojv@gmail.com; 3 Meteorology Research Unit, Swedish Meteorological & Hydrological Institute, SE 601 76 Norrköping, Sweden; christian.asker@smhi.se; 4 Institute of Family Medicine and Public Health, University of Tartu, 50090 Tartu, Estonia

**Keywords:** residential wood combustion, PM_2.5_, health impact assessment, economic valuation, burden of disease

## Abstract

**Highlights:**

**Public health relevance—How does this work relate to a public health issue?**

- Residential wood combustion is a major source of locally emitted PM_2.5_ in Nordic regions and contributes to population exposure even in relatively low-pollution environments.
- This study quantifies the mortality, morbidity, and economic burden attributable to PM_2.5_ from residential wood burning in northern Sweden.

**Public health significance—Why is this work of significance to public health?**

- The findings show that residential wood-smoke exposure is associated with measurable health impacts, including childhood asthma, COPD, cardiovascular disease, dementia, and years of life lost.
- Health-related costs attributable to residential wood combustion were estimated at approximately €7 million annually, indicating that substantial public health burdens can occur at relatively low exposure levels.

**Public health implications—What are the key implications or messages for practitioners, policy makers and/or researchers in public health?**

- Health impacts of residential wood combustion should be considered alongside climate and energy objectives when evaluating renewable heating strategies.
- Policies promoting cleaner heating technologies, replacement of older stoves, and improved combustion practices may provide significant public health benefits while supporting sustainable energy transitions.

**Abstract:**

Background: Residential wood combustion (RWC) is a major local source of fine particulate matter (PM_2.5_) in Nordic regions. We quantified the health impacts and economic costs of long-term exposure to PM_2.5_ from RWC in Västerbotten County, northern Sweden. Methods: High-resolution annual mean concentrations of PM_2.5_ from small-scale residential heating in 2023 were obtained from a national dispersion-modelling system. Population-weighted exposure was calculated by combining grid-level concentrations with home address coordinates for the resident population. Health impact assessment (HIA) followed the framework used in Nordic studies and the VALESOR project, applying a near-source PM_2.5_–mortality exposure–response function with a relative risk of 1.26 per 10 µg/m^3^ for natural-cause mortality and VALESOR default functions for morbidity (ischaemic heart disease, stroke, COPD, lung cancer, type 2 diabetes, dementia, childhood asthma, cardiovascular hospital admissions). Attributable cases and years of life lost (YLL) were estimated, and the economic valuation used VALESOR unit costs for Sweden to derive central, low and high damage-cost estimates. Results: The population (n = 280,742)-weighted mean RWC-PM_2.5_ exposure was 0.16 µg/m^3^ (interquartile range 0.10–0.21). This was associated with an estimated 30 years of life lost (YLL) per year from natural-cause mortality, plus incident morbidity of approximately 0.3 lung cancer cases, four childhood asthma cases, three COPD cases, three IHD events, one stroke, two dementia cases, one diabetes case and 0.8 cardiovascular hospital admissions annually. The total annual health-related cost attributable to RWC-PM_2.5_ was about €7.0 million (95% uncertainty range €4.6–9.4 million), dominated by mortality and disutility costs. Conclusions: PM_2.5_ concentrations from residential wood burning are associated with health impacts costing multi-million Euros in a Nordic region, supporting policies to reduce emissions from residential wood heating and to promote cleaner heating alternatives.

## 1. Introduction

Ambient fine particulate matter (PM_2.5_) is recognised as one of the leading environmental risk factors for premature mortality worldwide. Global burden-of-disease assessments and related modelling studies have shown that long-term exposure to PM_2.5_ contributes to millions of deaths annually and to substantial losses of life expectancy, even in high-income countries with comparatively low mean concentrations [1,2,3]. In response to this accumulating evidence [4], the World Health Organization (WHO) has issued increasingly stringent air-quality guidelines, most recently recommending an annual PM_2.5_ guideline value of 5 µg/m^3^ and emphasising that there is no apparent safe threshold [2].

A growing body of cohort evidence indicates that health risks from PM_2.5_ may vary by the source and mixture. Studies from Europe and North America suggest that near-source or locally emitted PM_2.5_, which is often dominated by traffic, residential combustion and other primary emissions, may be associated with higher mortality risks per 10 µg/m^3^ than more regionally uniform secondary particles [5,6,7]. In Swedish and Nordic health-impact assessments (HIAs), exposure–response functions (ERFs) corresponding to relatively steep “near-source” risk estimates have therefore been adopted [8,9,10], building on source-apportioned analyses such as those by Thurston et al. [6] and related low-exposure cohort work.

Residential wood combustion (RWC) is an important local source of primary PM_2.5_ in many temperate and cold-climate regions [11,12]. In the Nordic countries, where forests are abundant, and wood heating is culturally and historically embedded, RWC is among the dominant contributors to wintertime PM_2.5_ in cities such as Oslo, Helsinki, Copenhagen and several Swedish urban areas. Dispersion-modelling studies show that RWC can contribute from fractions of a microgram up to several µg/m^3^ to the annual mean PM_2.5_, depending on the appliance stock, combustion practices and local meteorology.

Epidemiological and toxicological evidence supports concern about the health effects of woodsmoke. Observational studies in high-income countries have linked indicators of RWC exposure to increased respiratory symptoms, reduced lung function and emergency visits for asthma, particularly among children and older adults [13]. Long-term exposure to PM_2.5_ from residential wood burning and other local combustion sources has also been associated with dementia incidence in longitudinal cohorts [14]. In addition, indoor measurements in wood-stove homes show that stove operation can substantially elevate the indoor PM_2.5_ concentrations, and stove-replacement programmes such as the Libby change-out in Montana have demonstrated sizeable reductions in indoor levels when old stoves are replaced by modern low-emission models [15].

In Sweden and other Nordic countries, RWC continues to be promoted in some contexts as a renewable and climate-friendly heating option, particularly in rural areas, forest-rich regions and during periods of energy-price volatility. However, while the climate implications of replacing fossil fuels with biomass have been extensively discussed, the associated local health impacts have received comparatively less attention in energy-policy debates. Residential wood combustion emits substantial quantities of primary PM_2.5_ and other pollutants that can adversely affect human health, even in regions that otherwise meet air-quality standards. Recent Nordic assessments have suggested that woodsmoke may account for a considerable share of the remaining air-pollution-related health burden under low ambient pollution conditions [10,11,12]. This highlights a potential policy trade-off: measures intended to support renewable energy use and energy security may simultaneously generate negative health externalities unless emissions are adequately controlled.

Although several studies have quantified the air-quality impacts of residential wood combustion in Nordic countries, fewer have translated these exposures into comprehensive estimates of the disease burden and economic costs at the regional level. Moreover, there is limited evidence from sparsely populated northern regions where wood heating remains common and is often regarded as a sustainable energy source. Assessing these health externalities is important for designing policies that balance climate, energy-security and public-health objectives and for identifying opportunities to achieve co-benefits through cleaner heating technologies and improved emission controls.

Although previous Nordic studies have investigated the contribution of residential wood combustion to ambient PM_2.5_ concentrations and associated health burdens, relatively few studies have combined high-resolution source-specific exposure modelling with a comprehensive assessment of mortality, morbidity, and economic costs at the regional level. Furthermore, the evidence remains limited for sparsely populated northern regions, where wood heating is common and frequently promoted as a renewable energy source. This study addresses this gap by providing a detailed health and economic assessment of PM_2.5_ originating from residential wood combustion in northern Sweden.

HIA is a widely used tool for translating population exposures into estimates of attributable mortality, morbidity and economic costs. It combines exposure modelling, baseline health statistics and ERFs to estimate excess cases and years of life lost under different scenarios. Recent European work, including the VALESOR project, has further integrated economic valuation of health impacts into this framework, allowing the comparison of air-pollution damage costs with the costs of mitigation measures.

Against this background, the present study applies a detailed dispersion-modelling and HIA framework to estimate the health effects and associated economic costs of long-term exposure to PM_2.5_ from residential wood combustion in Västerbotten County, northern Sweden. Using a near-source PM_2.5_–mortality ERF corresponding to a relative risk of 1.26 per 10 µg/m^3^, derived from source-related analyses of U.S. cohorts and adopted in Nordic HIAs [9,10,16], and a set of established ERFs for multiple morbidity outcomes [4], we quantify the annual burden of mortality and incident disease attributable to woodsmoke PM_2.5_ and monetise this burden using the VALESOR valuation framework [17].

Our aim is to provide locally relevant evidence to guide air-quality, energy and climate-policy decisions concerning residential wood burning in a relatively low-pollution Nordic region and to quantify the health externalities that should be considered when evaluating renewable heating strategies.

## 2. Methods

### 2.1. Study Area and Population

The study area is Västerbotten County in northern Sweden, with 280,742 inhabitants during the study year of 2023. Long-term exposure to PM_2.5_ from RWC was assessed and linked to health outcomes in the resident population. Baseline demographic data (population by age and sex) and baseline mortality and morbidity rates were obtained from Swedish national registers and harmonised to the spatial units used in the exposure assessment, as in previous Swedish and Nordic burden-of-disease assessments [9,10].

Baseline mortality was restricted to adults (≥30 years) in line with the underlying cohort evidence used for the ERFs [4].

### 2.2. Air Pollution Exposure Assessment

#### Dispersion Modelling

The annual average PM_2.5_ concentrations for 2023 were obtained from the national high-resolution dispersion-modelling system developed by the Swedish Meteorological and Hydrological Institute (SMHI) [18]. This modelling framework combines (i) a regional Eulerian chemical transport model (MATCH) over Europe and Sweden, (ii) urban-scale Gaussian dispersion modelling (NG2M) within the CLAIR toolbox, and (iii) a street-canyon model (OSPM) for traffic-influenced street segments.

MATCH simulations include anthropogenic and natural emissions (e.g., forest fires from CAMS-GFAS) at 0.1° resolution over Europe and 5 km over Sweden. To reduce bias, the modelled concentrations are statistically adjusted against hourly and daily observations from the Swedish monitoring network, and the residual bias field is interpolated and applied across the country. High-resolution urban-scale calculations are then performed for 10 × 10 km tiles using NG2M/CLAIR, where emissions are represented as point, line, area and gridded sources. Source apportionment is provided for several sectors, including small-scale residential heating (GNFR sector C2), emitted on a 100 m grid.

For this study, the annual mean contribution of PM_2.5_ from small-scale residential heating (RWC) was extracted from the model output for all grid cells intersecting Västerbotten County. The final exposure field thus represents the modelled annual mean PM_2.5_ from RWC at high spatial resolution (50 × 50 m, aggregated from the CLAIR output), expressed in µg/m^3^.

### 2.3. Population-Weighted Exposure

The population-weighted mean exposure to PM_2.5_ from RWC was calculated. For each grid cell *i*, the modelled concentration *Cᵢ* was combined with the resident population *Pᵢ*:

$$C_{\mathrm{pop}}=\frac{\sum_{i}C_{i}P_{i}}{\sum_{i}P_{i}}.$$

This metric assigns more weight to emissions in densely populated areas and represents the average exposure of the resident population to RWC-related PM_2.5_.

### 2.4. Health Impact Assessment

We applied a quantitative HIA framework similar to that used by Orru et al. (2022) [10] and in other European assessments, combining (i) modelled long-term exposure to PM_2.5_ from RWC, (ii) baseline mortality and morbidity rates, and (iii) ERFs derived from epidemiological studies and compiled in the VALESOR project [17].

#### 2.4.1. Health Outcomes

Health impacts were quantified for natural (non-accidental) all-cause mortality in adults and for a set of chronic morbidity endpoints for which VALESOR provides default PM_2.5_ ERFs and baseline rates (e.g., ischaemic heart disease, stroke, COPD, lung cancer, type 2 diabetes, dementia, childhood asthma) [17]. The selection and definition of endpoints follow the VALESOR health-endpoint set implemented in the VALESOR-Air tool and described in its methodological guidelines.

#### 2.4.2. Exposure–Response Functions

For natural all-cause mortality, we used a log-linear ERF corresponding to a hazard ratio (HR) of 1.26 (95% CI 1.19–1.34) per 10 µg/m^3^ increase in long-term PM_2.5_ exposure, consistent with near-source PM_2.5_ effect estimates from analyses of the American Cancer Society cohort [6] and adopted in recent Nordic HIAs [9,10]. This reflects the higher toxicity attributed to locally emitted (primary) PM_2.5_ compared with more regionally uniform secondary particles.

For a change in concentration ΔC (µg/m^3^), the relative risk (RR) is

$$RR=exp(\beta \;\Delta C),\beta =\frac{ln(1.26)}{10}.$$

For morbidity endpoints, we used the default PM_2.5_ ERFs implemented in VALESOR, which were selected based on causality assessments and systematic reviews (including WHO HRAPIE and subsequent evidence syntheses) and documented in VALESOR. Each ERF specifies the exposure range and age groups for which the endpoint is applicable; in this HIA, we applied them within those ranges.

The ERFs applied in this study were derived primarily from large cohort studies conducted in North America and Europe and subsequently adopted in Nordic and European HIA frameworks. The rationale for using these functions is that they represent the strongest available epidemiological evidence linking long-term PM_2.5_ exposure to mortality and morbidity outcomes and provide a consistent basis for comparison with previous burden-of-disease assessments. Furthermore, several recent cohort studies have reported associations between PM_2.5_ and adverse health outcomes at concentrations well below current European air-quality standards, supporting the use of these relationships in relatively low-exposure settings.

Nevertheless, transferring ERFs across geographical regions introduces uncertainty. The composition and toxicity of PM_2.5_ may differ according to the emission sources, atmospheric conditions and population characteristics. In particular, the mortality ERF used in this study originates from studies of near-source PM_2.5_, including mixtures influenced by traffic and other combustion sources, whereas PM_2.5_ from residential wood combustion may differ in chemical composition and health effects. In addition, differences in healthcare systems, socioeconomic conditions, baseline disease rates and population susceptibility may influence the generalisability of reported risk estimates. As a result, the true health effects of residential woodsmoke exposure in northern Sweden may be either lower or higher than those estimated in the present assessment.

The mortality ERF selected for this study is higher than the pooled risk estimates commonly reported for total ambient PM_2.5_. For example, recent meta-analyses of cohort studies have reported relative risks for all-cause mortality of approximately 1.08 per 10 µg/m^3^ increase in long-term PM_2.5_ exposure. In contrast, the estimate used here (RR = 1.26) originates from source-apportioned analyses focusing on near-source and primary combustion-related PM_2.5_ and has been adopted in several Nordic HIAs. The underlying rationale is that locally emitted primary particles from combustion processes may exhibit higher toxicity than regional secondary particles, resulting in steeper exposure–response relationships.

#### 2.4.3. Attributable Cases and Uncertainty

Attributable fractions (AF) and numbers of cases were calculated using standard HIA formulas, as in the WHO AirQ+ and the VALESOR methodological documentation. For each endpoint and stratum (age/sex):

$$\begin{array}{l} AF=\frac{RR-1}{RR},\\ \mathrm{Attributable}\;\mathrm{cases}=AF\times I_{0}\times N, \end{array}$$

where *I*_0_ is the baseline incidence (or mortality) rate, and *N* is the exposed population. For mortality, we additionally estimated years of life lost (YLL) by multiplying the attributable deaths by the age- and sex-specific remaining life expectancy.

Uncertainty in health impacts was characterised by propagating the 95% confidence interval (CI) of the ERFs. Lower and upper RR bounds were used to derive low and high AF and attributable cases, holding exposure and baseline rates constant. This yielded central, low and high estimates of health impacts.

### 2.5. Economic Valuation

#### Valuation Framework

Economic valuation followed the impact-pathway approach used in VALESOR and its predecessor model ALPHA-RiskPoll, which links emissions to exposure, health outcomes and then monetary values for each endpoint. We quantified annual health-damage costs associated with PM_2.5_ from RWC in terms of

- Mortality: value of life years lost (VOLY);
- Morbidity: value of a statistical case (VSC), combining cost-of-illness (COI) and disutility (quality-of-life loss).

For each endpoint *k*, the total annual damage (Dₖ) is

$$D_{k}={\mathrm{Attributable}\;\mathrm{cases}}_{k}\times {\mathrm{Unit}\;\cos t}_{k}.$$

Unit costs for Sweden were taken from the VALESOR default economic database (Table 1), which compiles and harmonises European COI studies and stated-preference valuation studies (VSL, VOLY, QALY-based valuations), adjusting to a common price year and currency and avoiding double-counting across components. The cost components include

- Direct medical costs (health-care utilisation),
- Indirect costs (productivity losses in paid and unpaid work),
- Disutility (loss of welfare/quality of life), monetised via willingness-to-pay.

Costs are reported in million Euros (Euro 2023) after currency conversion and inflation adjustments made according to the VALESOR tool. To avoid conveying unwarranted precision, health-economic unit costs are presented rounded to the nearest thousand Euros, while calculations were performed using the original values contained in the VALESOR database.

**Table 1:** Health economic costs used in the VALESOR tool for Sweden.

**Health Outcome**	**Total Morbidity Cost** **(€ Thousand/Case)**	**Health Care** **(€ Thousand)**	**Productivity Loss** **(€ Thousand)**	**Disutility** **(€ Thousand)**
Lung cancer	242	41	151	50
Childhood asthma	297	9	25	263
COPD	100	22	14	65
IHD events	190	12	3	175
Stroke	545	264	18	263
Diabetes	318	20	22	276
Dementia	554	288	41	226

### 2.6. Central, Low and High Valuation

For each endpoint we report the central, low and high economic estimates, consistent with the uncertainty structure in VALESOR. The central estimate uses the default unit-cost value for Sweden, whereas the low and high estimates draw on alternative valuation studies or parameter assumptions (e.g., lower/upper VOLY, alternative COI estimates) encoded as “low” and “high” unit-cost scenarios in the VALESOR database.

These valuation scenarios were combined with the central, low and high health-impact estimates derived from the ERF confidence intervals, yielding a range of plausible annual health-damage costs attributable to PM_2.5_ from RWC.

### 2.7. Methodological Considerations

Methodologically, we followed VALESOR recommendations to

- Avoid double-counting across overlapping endpoints (e.g., between incident chronic disease and related hospital admissions),
- Prefer incidence-based over prevalence-based COI and valuation where possible,
- Explicitly document major sources of uncertainty related to ERFs, exposure assessment and economic valuation.

This approach aligns the present HIA and valuation with the current European practice for assessing health and economic impacts of air pollution, while exploiting the high spatial resolution and source apportionment available from the national dispersion-modelling system.

## 3. Results

**Figure 1:**
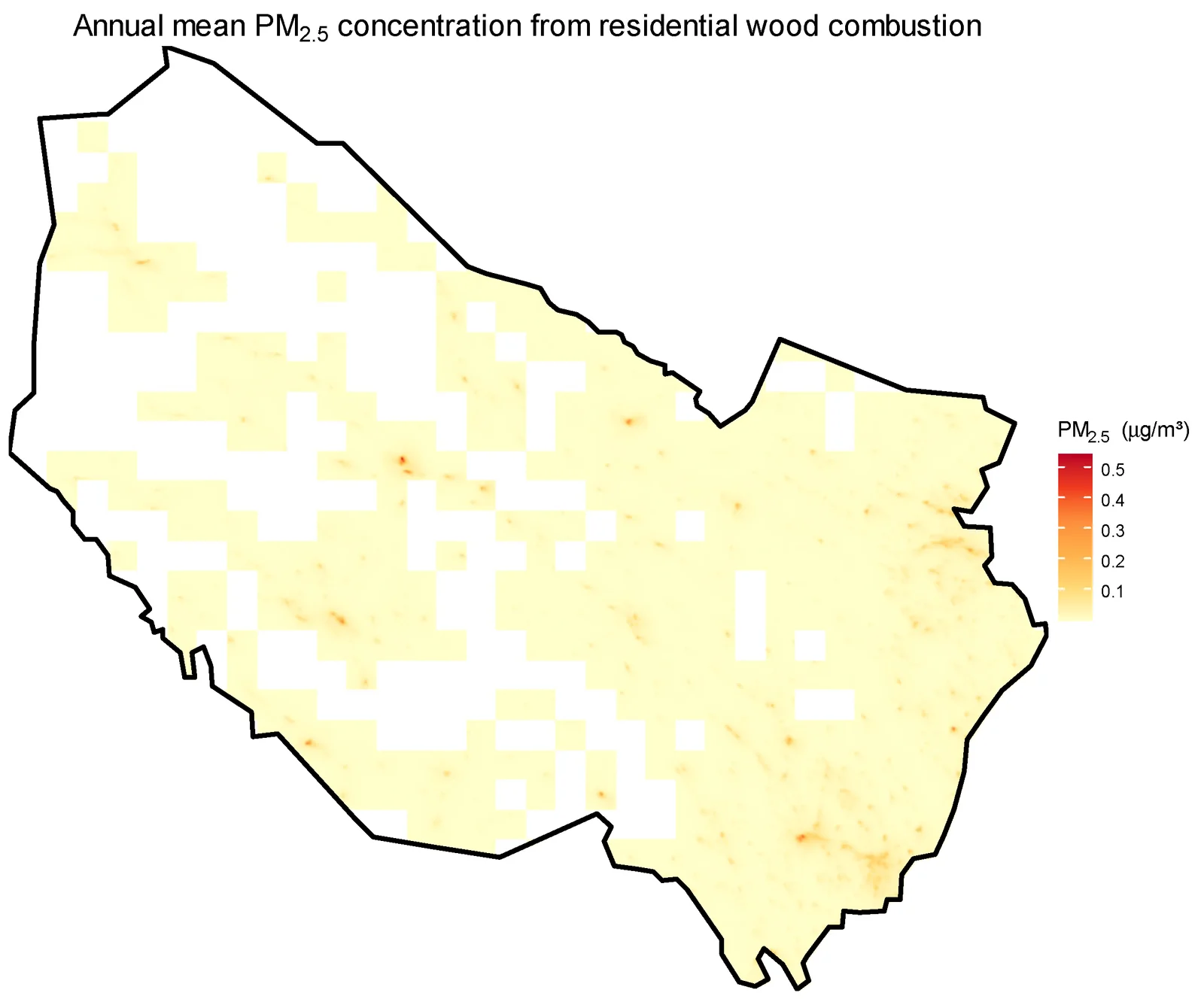
Modelled annual mean PM_2_._5_ concentrations attributable to residential wood combustion (RWC) in Västerbotten County, Sweden, during 2023. The map shows locally corrected annual average PM_2.5_ concentrations (µg/m^3^). Higher concentrations are indicated by darker colours.

In the study area (n = 280,742 inhabitants), the population-weighted annual mean exposure to PM_2.5_ from residential woodsmoke was 0.16 µg/m^3^ (median 0.16 µg/m^3^; IQR 0.10–0.21 µg/m^3^). The spatial distribution of annual mean PM_2_._5_ concentrations attributable to residential wood combustion (RWC) across Västerbotten County is shown in Figure 1.

### 3.1. Health Effects

Among the morbidity outcomes, childhood asthma and COPD accounted for the highest number of excess cases, while dementia contributed the largest share of morbidity-related economic costs. Although uncertainty intervals varied across outcomes, all estimates consistently indicated a non-zero health burden associated with PM_2.5_ from residential wood combustion.

**Figure 2:**
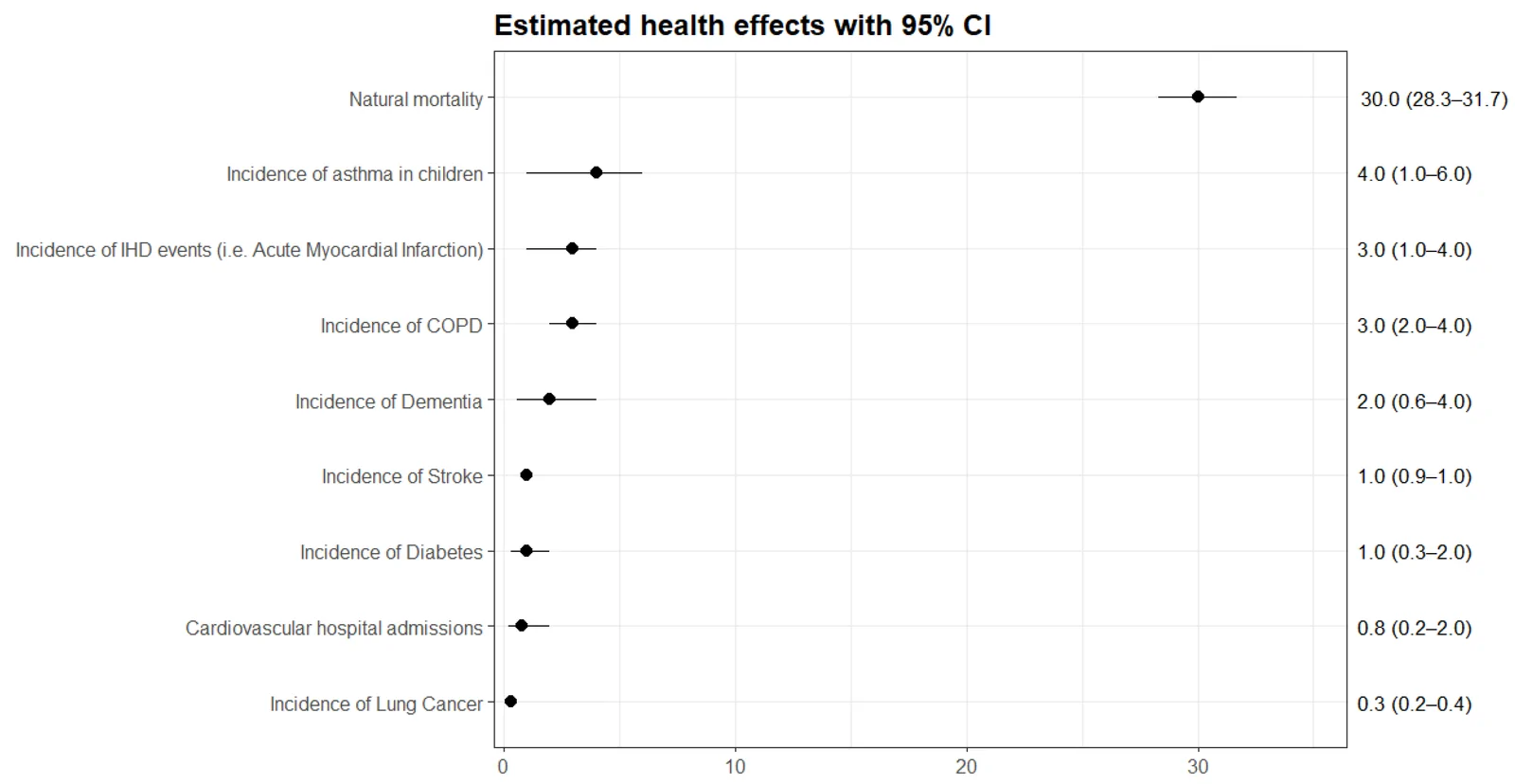
Estimated annual number of cases.

The estimated health impacts on both mortality and morbidity attributable to PM_2.5_ from woodsmoke is presented in Figure 2. The central estimate for natural-cause mortality was 30 years of life lost (YLL) per year in the county. In addition, the model yielded approximately 15 incident non-fatal cases of disease per year distributed across several chronic conditions. The largest single contribution came from asthma in children, with an estimated four additional cases per year (95% CI 1–6). Among adults, the model suggested three incident COPD cases (95% CI 2–4), three incident ischaemic heart disease (IHD) events (95% CI 1–4), and two dementia cases (95% CI 0.6–4) annually attributable to woodsmoke PM_2.5_. For stroke and type 2 diabetes, the estimated excess was around one additional case per year each (diabetes 95% CI 0.3–2.0), while the excess lung cancer burden was small in absolute numbers (around 0.3 cases per year, 95% CI 0.2–0.4).

### 3.2. Economic Costs

**Figure 3:**
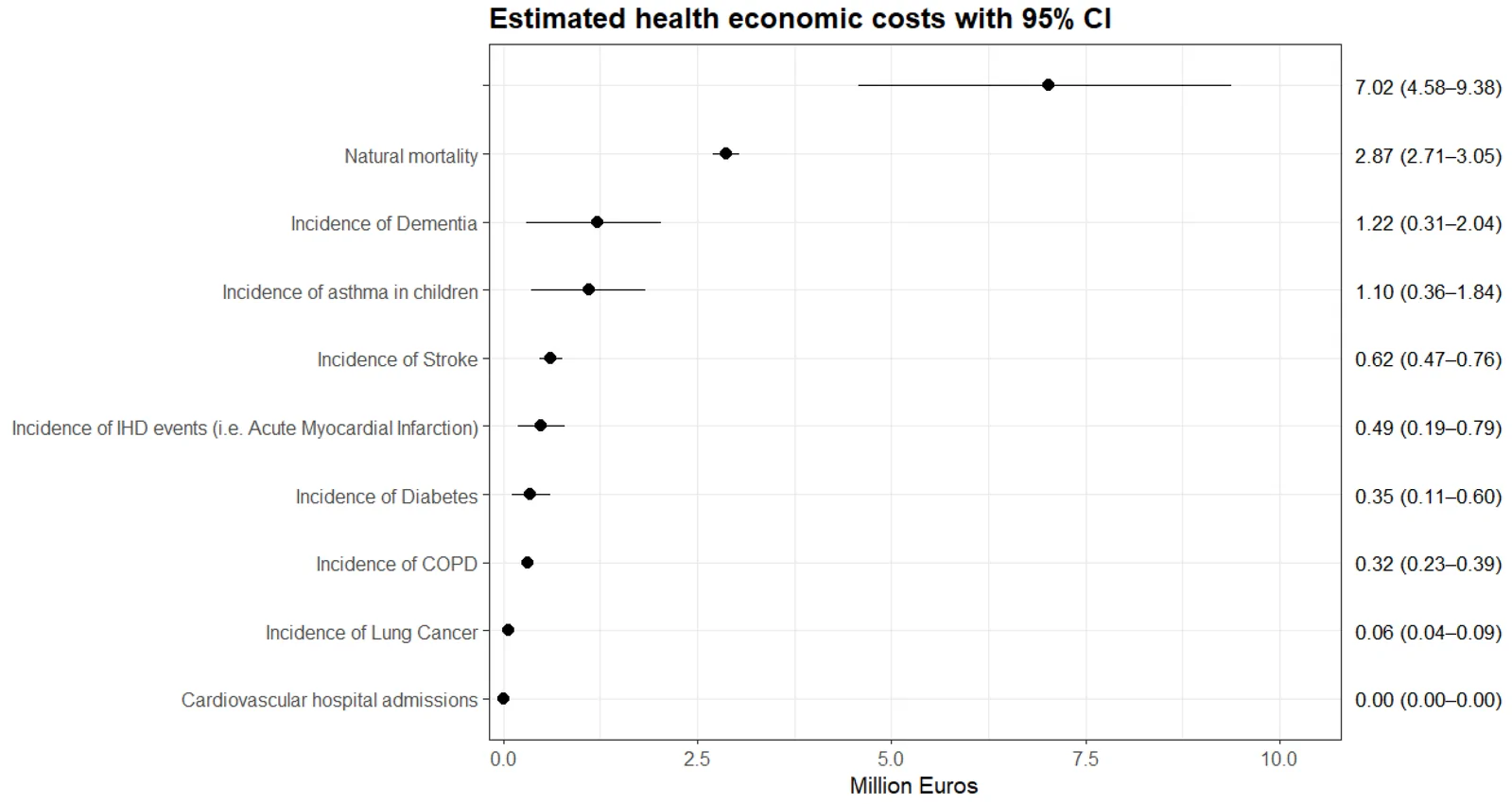
Estimated economic costs.

When these health outcomes were monetised in 2023 Euros (Figure 3), the total annual health-related cost of PM_2.5_ from residential woodsmoke in the county was estimated at €7.0 million (central estimate €7.02 million; 95% CI €4.58–9.38 million). This total reflects the sum of four cost components. The valuation of premature mortality (based on VOLY) contributed €2.87 million (41% of total). Disutility associated with morbidity, reflecting loss of quality of life among surviving cases, accounted for €2.74 million (95% CI €1.1–4.36 million) (39%). Direct healthcare costs (inpatient and outpatient care and pharmaceuticals) contributed €1.10 million (95% CI €0.47–1.67 million), while productivity losses amounted to about €0.32 million (95% CI €0.14–0.49 million), i.e., 4–5% of the total.

From the perspective of specific health outcomes, the largest single monetary contribution came from natural-cause mortality (€2.87 million per year). Among non-fatal conditions, dementia and childhood asthma were the most important, with central cost estimates of €1.22 million (95% CI €0.31–2.04 million) and €1.10 million (95% CI €0.36–1.84 million), respectively. Stroke, IHD events, diabetes, COPD and lung cancer each contributed smaller amounts (between approximately €0.06 and €0.62 million annually).

## 4. Discussion

The present HIA shows that a 0.16 µg/m^3^ (IQR 0.10–0.21) population-weighted exposure to PM_2.5_ from residential wood combustion is associated with a measurable burden of disease and substantial economic costs in Västerbotten. We estimated about 30 YLLs per year together with a number of incident cases of childhood asthma, COPD, ischaemic heart disease (IHD) events, stroke, dementia, diabetes and lung cancer attributable to RWC-related PM_2.5_. When monetised using the VALESOR framework, the total annual cost was in the order of several million Euros, dominated by the valuation of premature mortality and morbidity-related disutility.

Our results are broadly consistent with earlier Nordic assessments of RWC. Orru et al. (2022) estimated that RWC-PM_2.5_ contributes 0.5–2.8 µg/m^3^ to urban background concentrations in four Nordic city regions and causes between tens and hundreds of premature deaths annually, depending on the population size and exposure [10]. Although our county-wide exposure is smaller, the pattern of impacts, with natural-cause mortality as the single largest contributor and non-fatal cardio-respiratory morbidity adding appreciably to the burden, is similar. When standardised by population size and exposure level, our mortality estimates are comparable also to those obtained in health-impact assessments for southern Sweden and national-level analyses using similar ERFs [8,9].

To quantify mortality, we applied an ERF corresponding to a relative risk of 1.26 per 10 µg/m^3^ increase in PM_2.5_, reflecting near-source primary PM_2.5_ effects, as reported in source-apportioned analyses of the American Cancer Society cohort and in related low-exposure studies [6,7,13]. This choice is in line with evidence that PM_2.5_ from primary combustion sources, such as traffic and wood burning, may be more strongly associated with mortality than secondary particles.

An additional consideration is the transferability of exposure–response functions across regions and exposure settings. The ERFs used in this study were developed from large epidemiological cohorts outside northern Sweden and, therefore, assume that the health effects per unit exposure are broadly comparable between populations. This assumption is supported by evidence that PM_2.5_-related health effects occur even at relatively low concentrations and by the widespread use of these ERFs in European and Nordic burden-of-disease assessments. However, uncertainty remains regarding whether particles from residential wood combustion in northern Scandinavia have the same toxicity as the source mixtures represented in the underlying cohort studies.

The choice of mortality ERF deserves particular consideration. Large meta-analyses and assessments underpinning international burden-of-disease studies have generally reported lower all-cause mortality risks associated with long-term PM_2.5_ exposure, often around 1.06–1.10 per 10 µg/m^3^. By comparison, the RR of 1.26 used in the present analysis represents the upper end of estimates currently employed in health impact assessments. We selected this value because it was derived from analyses of source-related and near-source PM_2.5_ and has been adopted in previous Nordic assessments seeking to capture the potentially higher toxicity of primary combustion particles.

However, the extent to which such findings can be transferred to residential woodsmoke exposure in northern Sweden remains uncertain. Woodsmoke particles differ from traffic-related particles and other combustion mixtures with respect to their chemical composition, particle size distribution, oxidative potential and seasonal exposure patterns. Moreover, the population-weighted exposure levels in the present study were substantially lower than those observed in many cohorts used to derive exposure-response functions. Although increasing evidence suggests that adverse health effects occur even at very low PM_2.5_ concentrations, uncertainty remains regarding the slope of the exposure–response relationship for woodsmoke at these concentrations. Consequently, the estimated burden presented here should be interpreted as an estimate based on a precautionary yet plausible risk assumption rather than a precise prediction of attributable disease.

Future studies would benefit from source-specific epidemiological analyses of residential woodsmoke exposure and health outcomes in Nordic populations, which could reduce the uncertainty regarding both the magnitude and shape of the exposure–response relationship under low-concentration conditions.

Our inclusion of several non-fatal outcomes aligns with the broader epidemiological and toxicological literature. Long-term PM_2.5_ exposure has been linked not only to all-cause and cardio-respiratory mortality but also to incident cardiovascular disease, COPD, type 2 diabetes, lung cancer, dementia and other neurological outcomes [4]. Epidemiological studies in Nordic and other high-income settings have linked indicators of residential wood burning to increased respiratory symptoms, reduced lung function and emergency visits for asthma, particularly among children [13]. Cohort evidence also suggests that long-term exposure to local combustion-related PM_2.5_ may contribute to dementia incidence [14]. Our finding showed that childhood asthma and dementia together account for a large share of the monetised burden.

### 4.1. Strengths and Limitations

Several limitations and sources of uncertainty should be noted. First, by adopting the near-source RR of 1.26 per 10 µg/m^3^ for mortality, we implicitly assume that the toxicity of PM_2.5_ from residential wood burning in Västerbotten is comparable to the primary PM_2.5_ mixtures in the underlying cohorts. If local woodsmoke particles are less toxic, our mortality estimates would be biased upwards; if they are more toxic, our estimates may instead be conservative.

Second, exposure was characterised using annual average ambient concentrations at the residential location, aggregated to a population-weighted mean. This is standard practice in HIAs and consistent with the spatial resolution of most long-term PM_2.5_ cohort studies. However, it does not capture short-term peaks associated with evening firing, inversions or cold spells, nor does it reflect time–activity patterns that may lead to higher exposures for subgroups who spend more time at home during stove operation. Spatial models suggest that using coarser grids tends to underestimate the population exposure to primary particles from sources such as RWC, and therefore, the high resolution of our air pollution dispersion model is a strength of this study.

Third, we did not include indoor emissions from stoves and fireplaces, although these may substantially elevate personal exposure in wood-burning homes. Indoor measurements in wood-stove homes have shown that stove operation can markedly increase indoor PM_2.5_ concentrations and that replacement of older stoves can substantially reduce indoor exposure [15]. Indoor exposure to emissions from solid-fuel combustion is also recognized as an important contributor to respiratory and cardiovascular health effects [4]. However, incorporating indoor exposures into an ambient-based HIA is methodologically challenging, because personal exposure reflects both indoor and outdoor sources, and combining these pathways may introduce double counting of exposure and health impacts [17]. Consequently, our estimates should be interpreted as representing only the ambient outdoor component of the health burden attributable to residential wood combustion.

Fourth, the economic valuation involves normative choices and is sensitive to parameter assumptions. We used a VOLY-based approach for mortality and VSC-based valuations for morbidity, supplemented with components for healthcare costs, productivity losses and disutility, following the VALESOR framework. Similar approaches have been applied in European and global assessments of air-pollution-attributable disease burden. Nevertheless, the valuation of intangible welfare losses, such as reduced quality of life in dementia or chronic asthma, is inherently uncertain and may vary with societal preferences.

Finally, our analysis treated the population as homogeneous. We did not explicitly model socioeconomic or geographic inequalities in exposure and susceptibility, although previous work has shown that wood heating patterns and the distribution of older more polluting stoves can be socio-economically patterned within Nordic cities [9,10]. Vulnerable groups such as children, the elderly, people with chronic disease, and those with limited ability to invest in cleaner heating technologies may bear a disproportionate share of the health burden.

### 4.2. Policy Perspectives

From a policy perspective, our findings support integrating RWC more explicitly into air-quality management and climate-energy strategies. In many Nordic and other high-income regions, the promotion of biofuels as “carbon-neutral” has co-existed with relatively weak attention to local health impacts of wood burning. Measures such as phasing out old non-certified stoves, tightening emission standards, incentivising connections to district heating or heat pumps, and promoting best-practice firing (use of dry wood, avoiding smouldering) could yield co-benefits for both health and climate.

## 5. Conclusions

Our study indicates that PM_2.5_ from residential wood burning in a relatively small low-pollution county is associated with appreciable health impacts and economic costs. When set alongside the global and Nordic evidence on PM_2.5_ and health, these findings support continued efforts to tighten the regulation of residential wood combustion, promote cleaner heating alternatives and integrate air-quality considerations into climate and energy policy. At the same time, interventions must be designed with attention to energy affordability and cultural practices. For some rural households, wood remains an important back-up or primary heat source in the context of volatile electricity prices and concerns about energy security. Policies that combine financial support for stove replacement or fuel-switching with clear communication of the health risks and community engagement may be more acceptable and effective than purely restrictive measures. There is also a need to communicate that the “natural” origin of woodsmoke does not imply harmlessness, a misconception that persists in public debate despite strong evidence to the contrary.

## Data Availability

All data generated or analysed during this study are included in this published article.
